# Autoimmune glial fibrillary acidic protein astrocytopathy with anti-NMDAR and sulfatide-IgG-positive encephalitis overlap syndrome: A case report and literature review

**DOI:** 10.1097/MD.0000000000038983

**Published:** 2024-07-12

**Authors:** Ruo-mei Cui, Fu-rong Fan, Shou-hong Ma, Hua Li, Jin-chun Li, Yu Wen, Ming-wei Liu

**Affiliations:** a Department of Rheumatology, The First Hospital Affiliated to Kunming Medical University, Kunming, China; b Department of Emergency, The First Hospital Affiliated to Kunming Medical University, Kunming, China; c Department of Neurology, The six Hospital Affiliated to Kunming Medical University, Yuxi, China; d Department of Emergency, The Third People’s Hospital of Yunnan Province, Kunming, China; e Department of Emergency, Dali Bai Autonomous Prefecture People’s Hospital, Dali, China.

**Keywords:** Anti-NMDAR, Anti-Sulfatide-IgG, Autoimmune glial fibrillary acidic astrocytosis, overlap syndrome

## Abstract

**Rationale:**

Autoimmune glial fibrillary acidic protein (GFAP) astrocytopathy is a rare autoimmune disease of the central nervous system that affects the meninges, brain, spinal cord, and optic nerves. GFAP astrocytopathy can coexist with a variety of antibodies, which is known as overlap syndrome. Anti-NMDAR-positive encephalitis overlap syndrome has been reported; however, encephalitis overlap syndrome with both anti-NMDAR and sulfatide-IgG positivity has not been reported.

**Patient concerns:**

The patient was a 50-year-old male who was drowsy and had chills and weak limbs for 6 months. His symptoms worsened after admission to our hospital with persistent high fever, dysphoria, gibberish, and disturbance of consciousness. Positive cerebrospinal fluid NMDA, GFAP antibodies, and serum sulfatide antibody IgG were positive.

**Diagnoses:**

Autoimmune GFAP astrocytopathy with anti-NMDAR and sulfatide-IgG-positive encephalitis overlap syndrome.

**Interventions:**

In addition to ventilator support and symptomatic supportive treatment, step-down therapy with methylprednisolone (1000 mg/d, halved every 3 days) and pulse therapy with human immunoglobulin (0.4 g/(kg d) for 5 days) were used.

**Outcomes:**

After 6 days of treatment, the patient condition did not improve, and the family signed up to give up the treatment and left the hospital.

**Conclusions:**

Patients with autoimmune GFAP astrocytopathy may be positive for anti-NMDAR and sulfatide-IgG, and immunotherapy may be effective in patients with severe conditions.

**Lessons:**

Autoimmune GFAP astrocytopathy with nonspecific symptoms is rarely reported and is easy to be missed and misdiagnosed. GFAP astrocytopathy should be considered in patients with fever, headache, disturbance of consciousness, convulsions, and central infections that do not respond to antibacterial and viral agents. Autoimmune encephalopathy-related antibody testing should be performed as soon as possible, early diagnosis should be confirmed, and immunomodulatory therapy should be administered promptly.

## 1. Introduction

Autoimmune glial fibrillary acidic protein (GFAP) astrocytosis is a novel inflammatory autoimmune disease of the central nervous system. It can occur at all ages in acute or subacute conditions with various clinical manifestations. It is mainly characterized by inflammation of the meninges, brain parenchyma, spinal cord, or a combination of these sites.^[1]^ Patients often have a viral infection such as prodromus, with the main clinical manifestations being fever, headache, visual abnormalities, psychiatric disturbances, ataxia, movement disorders, and autonomic dysfunction, which are often accompanied by linear radial perivascular enhancement that marks the ventricles on nuclear magnetic resonance (MRI).^[1]^

Long et al^[2]^ reported that approximately 33.3% of patients with GFAP astrocytopathy have coexisting antibodies, such as N-methyl-d-aspartate receptor (NMDAR), Aquaporin-4 (AQP4), and myelin oligodendrocyte glycoprotein (MOG) antibodies, or their combinations.^[3–5]^ Thus, the coexistence of autoantibodies in patients with GFAP astrocytopathy is common, referred to as the overlap syndrome.^[6]^ However, there are no reports of GFAP astrocytopathy have been reported with positive NMDAR antibodies and sulfatide-IgG.

The purpose of this case report was to illustrate the diagnosis and treatment of a patient with GFAP astrocytopathy with anti-NMDAR and sulfatide-IgG-positive encephalitis overlap syndrome, to guide clinicians in understanding and treating this disease and to reduce missed diagnoses and misdiagnoses.

## 2. Case report

### 2.1. Ethics approval and consent to participate

Informed written consent was obtained from the patient for publication of this case report and accompanying images.

This study was reviewed and approved by the local ethics committee of the First Affiliated Hospital of the Kunming Medical University. The procedures were performed in accordance with the Helsinki Declaration of 1975 and were revised in 2000.

### 2.2. Medical history

A 50-year-old male patient treated with anti-infection therapy at a local hospital for drowsiness, chills, and limb weakness for half a month without remission was transferred to the emergency department of the First Affiliated Hospital of Kunming Medical University. Abdominal aortic aneurysm was diagnosed using abdominal aortic non-contrast scanning, enhanced scanning, and 3-dimensional reconstruction; thus, the patient was transferred to the Department of Vascular Surgery of the First Affiliated Hospital of Kunming Medical University for further treatment.

### 2.3. Past medical history

With diabetes mellitus for 5 months, the highest blood sugar was 8.1 mmol/L, oral metformin and glibenclamide, and the blood sandal was controlled at 0.7 mmol/L. There was no history of cardiovascular or cerebrovascular diseases, lung, camp, endocrine system, other main organ diseases, or infectious disease. No history of trauma, surgery, blood transfusion, allergy, or vaccination was reported.

### 2.4. Physical examination

Body temperature, pulse, respiration, and blood pressure were 37.8 °C, pulse: 124/min, respiration: 25 breaths/min, and 106/70 mm Hg, respectively. The patient’s general condition was slightly worse, the superficial lymph nodes were not large, and a fluctuating mass was palpable around the navel. Conscious, scored 15 on the Glasgow Coma Scale, fluent in speech, and with a normal orientation, numeracy, and memory. The 2 pupils had a diameter of approximately 3 mm, were sensitive to light reflex, the eyes moved freely in all directions, there was no nystagmus, the angle of the mouth did not deviate, the tongue was in the center, the neck was not resistant, the muscle tone of the limbs was normal, the muscle strength was grade 5, the tendon reflexes of the limbs were normal, Babinski syndrome was negative on both sides, and meningeal irritation reactions were negative. The acupuncture pain sensation was not reduced, and the bilateral finger-nose, heel–knee, and shin tests were stable and accurate. The Romberg (−) and mRS scores were 4, which did not indicate any apparent anxiety or depression.

### 2.5. Laboratory test data

Routine blood test: white blood cells 7.45 × 10^9^/L. The percentages of neutrophils, lymphocytes, eosinophils, red blood cells, Hg, and platelets were 82.0%, 16.2%, 0.20%, 4.19 × 10^12^/L, 126 g/L, and 301 × 10^9^/L, respectively. Blood biochemical test: sodium 141.82 mmol/L, chloride 95.14 mmol/L, alanine aminotransferase 109.00 IU/L, aspartate aminotransferase 6.50.00 IU/L, AST/ALT 1.47, direct bilirubin 18.4 µmol/L, creatinine 126.40 µmol/L, urea nitrogen 8.9 µmol/L. Infection-related protein assay: procalcitonin 0.46 ng/mL, hypersensitive C-reactive protein 94.00 mg/L. Cerebrospinal fluid routine test: colorless and transparent appearance, Paneth test (+), red blood cell count 49 × 10^6^/L, white blood cell count 1 × 10^6^/L, cerebrospinal fluid biochemical test: chlorine 117.10 mmol/L, total protein 0.530 g/L, albumin 498.8 mg/L. Quantitative assay of cerebrospinal fluid immunoglobulins: IgM, 4. 43 mg/L, IgG 70. 90 mg/L, IgA 7.91 mg/L. The results of cardiac enzymes, fungal smear, bacterial Gram stain, T cell spot test (T-SPOT), immunoglobulin and complement, Widal reaction, Weil-Felix test, tumor markers, urinalysis, systemic lupus erythematosus, rheumatoid-related antibodies, anticardiolipin antibodies, and antineutrophil cytoplasmic antibodies were negative. Tests for HIV, hepatitis, Epstein–Barr virus (EBV)-DNA, cytomegalovirus (CMV)-DNA, influenza A and B, and the new coronavirus were negative.

### 2.6. Imaging data

**Figure 1.**
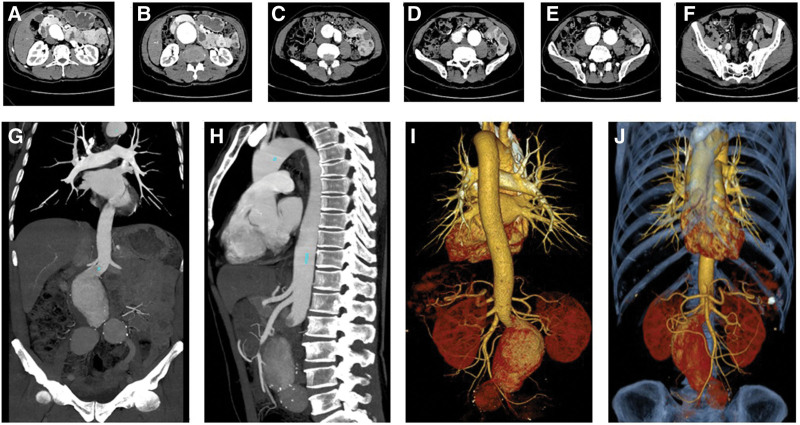
Abdominal vessels changes in enhanced CT findings. CT = computed tomography.

**Figure 2.**
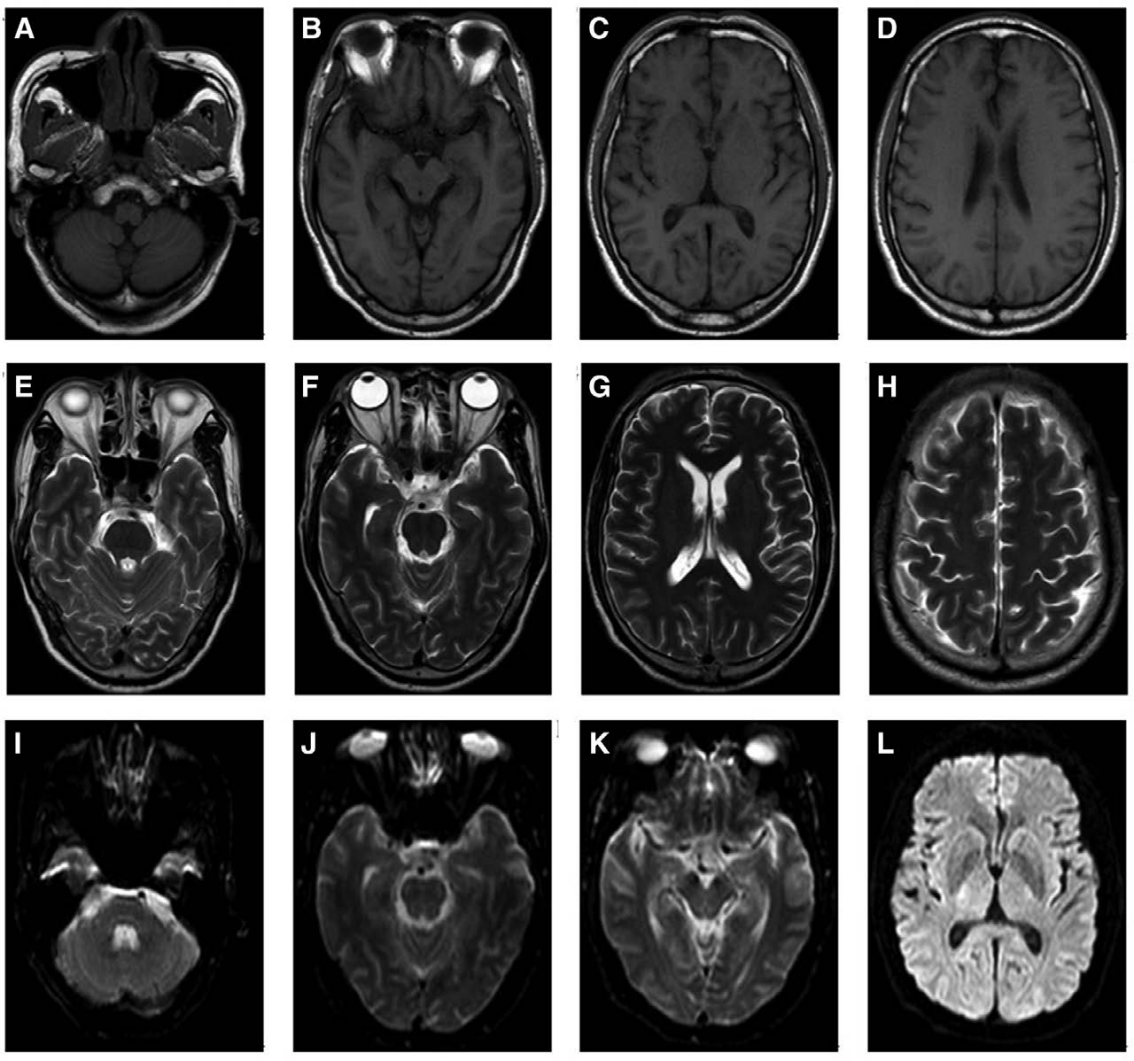
The changes in cranial MRI in the patient. MRI = nuclear magnetic resonance.

Abdominal aortic non-contrast computed tomography (CT) scan + enhanced scan + 3D reconstruction: abdominal aortic aneurysm with wall thrombosis (Fig. 1A–J), bilateral common and internal iliac aneurysms (Fig. 1A–J). Brain MRI non-contrast scan + enhanced scan: there was no abnormality in the morphology of the hemisphere, cerebellum, and brainstem on either side; no abnormal signal was found in the gray and white matter; and no abnormal enhancement was found in the enhanced scan (Fig. 2A–L). No abnormal signal was observed on SWI (Fig. 2A–L).

### 2.7. Diagnosis and treatment

April 12, 2023: According to clinical symptoms, laboratory treatment, brain MRI, and abdominal CT, the patient was initially diagnosed as: infection-related fever; abdominal aortic aneurysms with mural thrombosis; bilateral common iliac and internal iliac aneurysms. Intravenous infusion of ceftazidime (2 g) q12 was administered as anti-infection therapy.

April 14, 2023: Body temperature did not improve, patient’s condition worsened, and routine blood tests were performed: leukocyte count, 7. 45 × 10^9^/L; neutrophil percentage, 82%; and anti-infection therapy adjusted to meropenem 1 g every 8 hours.

April 15, 2023: The patient developed high fever, dysphoria, and gibberish, and there was no significant change in the cerebrospinal fluid (CSF) compared with the time of admission. Therefore, we considered the possibility of viral encephalitis and administered acyclovir as antiviral therapy.

April 19, 2023: The patient had aggravated consciousness impairment and was given endotracheal intubation and ventilator-assisted supportive treatment.

April 21, 2023: The patient developed symptomatic epilepsy and was administered valproate sodium antiepileptic therapy. The lumbar puncture cerebrospinal fluid was reexamined, and autoimmune imaginative encephalopathy-related antibody testing was performed.

**Figure 3.**
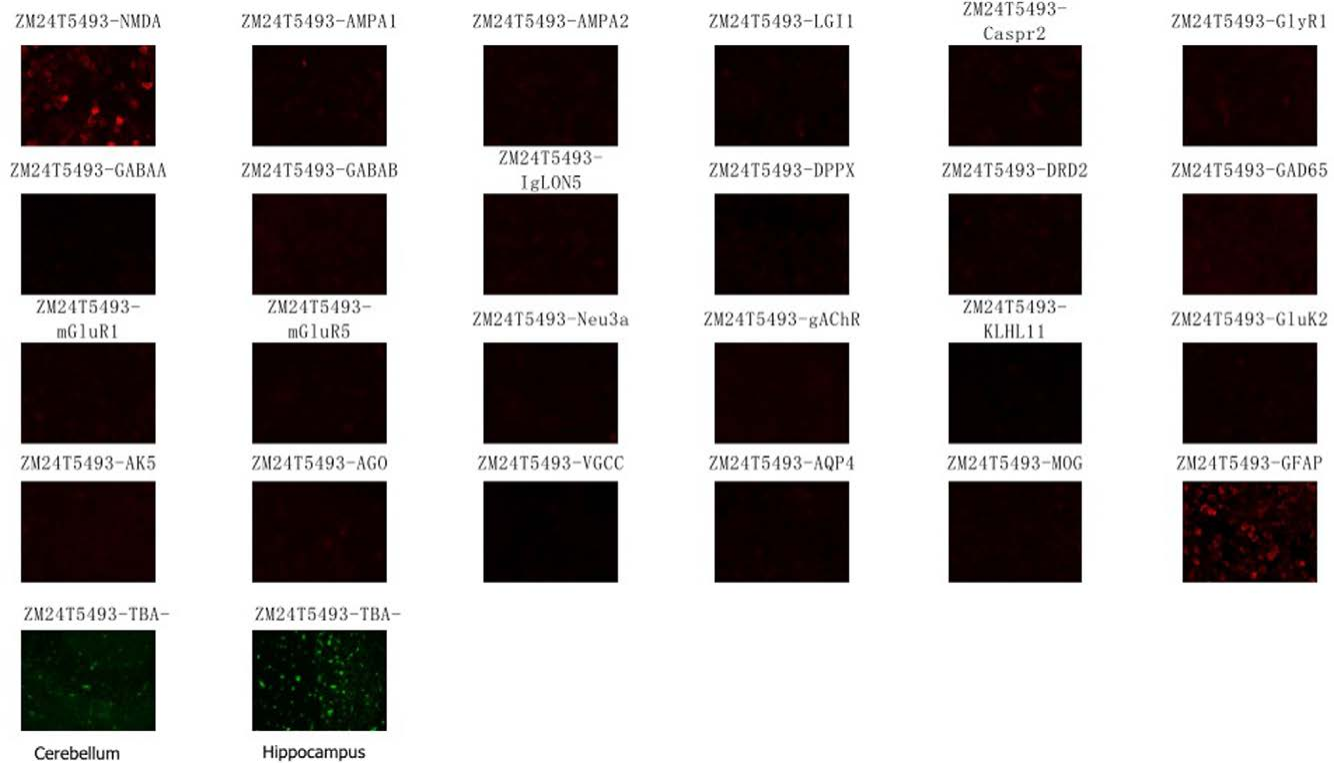
The changes autoimmune encephalitis antibodies in cerebrospinal fluid.

April 24, 2023: The detection results for antibodies related to autoimmune encephalopathy showed that cerebrospinal fluid was anti-NMDA antibody IgG positive (1:10), anti-TBAantibody(1:30) (Fig. 3), and anti-GFAP antibody: 1:32 (Fig. 3); serum autoimmune peripheral neuropathy was anti-sulfatide antibody IgG positive, while cerebrospinal fluid routine and biochemistry showed no apparent changes (Table 1). The diagnosis was adjusted to GFAP astrocytopathy with anti-NMDAR and sulfatide-IgG-positive encephalitis overlap syndrome. A step-down therapy with methylprednisolone (1000 mg/d, halved every 3 days) and pulse therapy with human immunoglobulin (0.4g/(kg d) for 5 days) was used.

**Table 1:** Measurement results of cerebrospinal fluid in patients.

Content	April 18, 2023	April 21, 2023
Cerebrospinal fluid routine		
Exterior	Clear and bright	Clear and bright
Pan experiment	Positive	Positive
White blood cell count (×10^6^/L)	1	4
Red blood cell count (×10^6^/L)	49	54
Biochemistry		
AST (U/L)	8.81	8.4
ALT (IU/L)	1.70	0.9
AST/ALT	5.18	9.33
ADA (U/L)	2.3	3.0
Glu (mmol/L)	3.75	4.74
MTP (g/L)	0.53	0.56
uMA (mg/L)	498.8	426.9
Cl (mmol/L)	117.10	137.7
CK (IU/L)	0.1	17
LDH (IU/L)	15	22
Immunoglobulin		
IgM (mg/L)	4.43	4.26
IgG (mg/L)	70.9	71.7
IgA (mg/L)	7.91	6.24

April 30, 2023: The patient’s symptoms did not improve, the Glasgow coma score was E3V2T, and the family signed up to give up the treatment and left the hospital.

### 2.8. Follow up

Two days after discharge, the patient became worse and died.

## 3. Discussion

Autoimmune GFAP astrocytopathy primarily affects the meninges, brain, spinal cord, and optic nerve, with fever, headache, encephalopathy, myelitis, and optic neuritis being the primary clinical symptoms.^[6–8]^ Most patients with autoimmune GFAP astrocytosis respond well to immunotherapy and have good prognosis. A Mayo Clinic study found that NMDAR-IgG was the most common coexisting antibody in patients with autoimmune GFAP, followed by AQP4-IgG.^[9]^ In contrast, studies in China have shown that AQP4-IgG is the most common coexisting antibody.^[10,11]^ In the present case, NMDAR-IgG was the most common antibody in patients with autoimmune GFAP, and patients with GFAP coexisted with sulfatide-IgG antibodies, which has not been previously reported. At present, the diagnostic significance of the overlap syndrome is unclear. There may be 2 or more immune mechanisms in patients with GFAP-A overlap syndrome, and there is no further difference in clinical symptoms between patients with overlap syndrome and those without overlap syndrome, except for the age of onset^[12]^; however, research has found that patients with overlapping antibodies have a poor response to immunotherapy, poor prognosis, and are more likely to relapse. In this patient, the response to corticosteroids combined with IVIG was poor and no obvious benefit was obtained, which may be related to the coexistence of GFAP with NMDAR-IgG and sulfatide-IgG antibodies.

Previous studies have found that anti-sulfatide antibodies can exist in a variety of diseases such as Guillain–Barré syndrome, chronic inflammatory demyelinating polyradiculoneuropathy, sensory and sensorimotor axonal neuropathy, multiple sclerosis, idiopathic thrombocytopenic purpura, autoimmune chronic active hepatitis, and diabetic neuropathy,^[13]^ and are mainly related to sensory neuropathy, peripheral neuropathy, axonal neuropathy, and primary demyelinating neuropathy in some patients.^[13,14]^ In the present case, we found that anti-sulfatide antibodies were present in autoimmune GFAP astrocytopathy and in patients with previous diabetes mellitus. However, no diabetic neuropathy was associated with anti-sulfatide antibodies. Sulfatide antibodies may be related to autoimmune GFAP astrocytopathy and may play a role in pathogenesis. Nevertheless, experimental evidence is lacking and needs to be further verified.

Patients with autoimmune GFAP astrocytopathy often have abnormal MRI of the brain and spinal cord, which is characterized by radial perivascular enhancement perpendicular to the lateral ventricles on enhanced MRI scans, with no abnormal DWI.^[15]^ Autoimmune GFAP astrocytopathy may involve the spinal cord and manifest on MRI with extensive longitudinal myelitis, mainly in the cervical and thoracic segments, and may be over 3 vertebral segments,^[15]^ characterized by central line-like enhancement on enhanced MRI scanning, sometimes punctate, patchy, and enhancement of the spinal pia mater.^[15]^ In this case, there was no abnormality in the non-contrast and enhanced brain MRI, and the inflammatory changes in the cerebrospinal fluid were mild, which led to misdiagnosis.

Anti-NMDAR encephalitis is one of the most common forms of autoimmune encephalitis, and is more common in young adults and children with acute or subacute onset, with typical clinical manifestations of abnormal mental behavior, memory impairment, seizures, and altered consciousness.^[16,17]^ Routine examination of the cerebrospinal fluid (CSF) in patients with anti-NMDAR encephalitis is normal, and the onset is mainly due to immunological abnormalities.^[16]^ In this case, the patient did not show the typical clinical symptoms of anti-NMDAR encephalitis, such as mental behavioral abnormalities and cognitive impairment, or the simple form of seizures, which are not as complex and diverse as the clinical manifestations of seizures caused by anti-NMDAR encephalitis.^[17]^ In addition, a cerebrospinal fluid test revealed inflammatory alterations, and the NMDAR antibody titer level was lower than that of the GFAP antibody, suggesting that the anti-NMDAR antibody was a concomitant antibody.

The disease can occur at all ages, with a median age of onset of >40 years and is slightly more common in women than in men.^[18]^ Most patients have clinical manifestations such as headache, fever, seizures, mental behavior abnormalities, and impaired consciousness, and 14% to 40% of patients have mild sensorimotor impairment related to the spinal cord.^[11,19]^ Other symptoms include visual disturbances, ataxia, intractable hiccups, peripheral nerve damage, and autonomic dysfunctions.^[11]^ Owing to these nonspecific symptoms and signs, autoimmune GFAP astrocytopathy is rare. Therefore, missed diagnoses and misdiagnoses are common. The patient presented with fever, malaise, and drowsiness upon admission. Anti-infectious therapy with ceftazidime for infectious fever should be administered, and the symptoms should worsen. High fever, dysphoria, gibberish, and central infection should be considered for meropenem, and acyclovir is ineffective. After multiple tests on admission, the patient’s cerebrospinal fluid immunoglobulin levels significantly increased, and the effectiveness of the antibacterial and antiviral treatments was unsatisfactory. Therefore, immune-related encephalitis, including autoimmune encephalitis and GFAP encephalitis, was considered, and further testing for autoimmune encephalopathy-related antibodies revealed GFAP antibodies in the cerebrospinal fluid. On the basis of the patient’s symptoms and signs, GFAP encephalitis was diagnosed and treated with methylprednisolone and immunoglobulin.

Currently, there is no unified diagnostic standard for the diagnosis of GFAP-A encephalitis. The main diagnostic mode for GFAP-A encephalitis is based on clinical manifestations, imaging findings, and positive detection of GFAP-A antibodies in the cerebrospinal fluid. Usually, if a patient presents with the following conditions that cannot be explained by other diseases, GFAP-A needs to be tested: clinical manifestations include meningoencephalitis, encephalitis, meningitis, meningoencephalomyelitis, and myelitis; MRI shows characteristic vascular enhancement. Further diagnosis requires a lumbar puncture examination to detect GFAP-A antibodies in the cerebrospinal fluid. However, currently only 40% to 50% of patients have typical cranial MRI manifestations,^[20]^ and 56% to 80% of patients have abnormal manifestations, such as elevated white blood cells and decreased glucose levels in the cerebrospinal fluid.^[21,22]^ At present, the internationally recognized standard for the diagnosis of autoimmune GFAP astrocytosis is GFAP-IgG positivity, and it is believed that positive cerebrospinal fluid anti-GFAP antibodies have a higher clinical value in the diagnosis of this disease.^[9]^ Although the patient’s head MRI showed no abnormalities, the had symptoms of encephalitis, such as fever, headache, coma, and convulsions, and the cerebrospinal fluid GFAP-A antibody was positive. These results support a diagnosis of GFAP-A.

## 4. Strengths and limitations

### 4.1. Strengths

Autoimmune GFAP astrocytopathy can coexist with NMDAR and sulfatide-IgG antibodies, and early immunomodulatory therapy may be effective.

### 4.2. Limitations

Autoimmune GFAP astrocytopathy can coexist with NMDAR and sulfatide-IgG antibodies. The symptoms are nonspecific and can easily be misdiagnosed. In addition, whether the positive sulfatide-IgG antibody results were due to peripheral neuropathy caused by diabetes remains to be determined. The patient’s cerebrospinal fluid was GFAP-A positive and showed symptoms and signs of encephalitis. However, there were no abnormalities on cranial MRI, and no abnormalities were found on routine and biochemical examinations of the cerebrospinal fluid. Therefore, anti-NMDAR encephalitis has not been completely excluded.

## 5. Conclusions

Autoimmune GFAP astrocytopathy is rarely reported, and its symptoms are nonspecific and can be easily missed and misdiagnosed. Autoimmune GFAP astrocytopathy can coexist with NMDAR and sulfatide-IgG antibodies, which has not been reported. In addition to considering central infection with antibacterial and viral infection treatments, patients with fever, headache, consciousness disorders, convulsions, and autoimmune encephalopathy should be considered, and antibody testing should be performed as soon as possible to enable early diagnosis and timely immunomodulatory therapy.

## Acknowledgments

We thank the editorial team of Home for Researchers (www.home-for-researchers.com) for their language-editing services.

## Author contributions

**Data curation:** Ruo-mei Cui, Shou-hong Ma, Jin-chun Li, Ming-wei Liu.

**Formal analysis:** Ruo-mei Cui, Hua Li, Jin-chun Li.

**Investigation:** Ruo-mei Cui, Fu-rong Fan, Hua Li, Jin-chun Li, Yu Wen, Ming-wei Liu.

**Project administration:** Ruo-mei Cui, Hua Li, Jin-chun Li.

**Resources:** Ruo-mei Cui, Fu-rong Fan, Hua Li, Jin-chun Li, Yu Wen, Ming-wei Liu.

**Supervision:** Ruo-mei Cui, Fu-rong Fan, Hua Li, Jin-chun Li, Ming-wei Liu.

**Validation:** Ruo-mei Cui, Shou-hong Ma, Hua Li, Jin-chun Li.

**Funding acquisition:** Fu-rong Fan, Shou-hong Ma, Jin-chun Li, Yu Wen.

**Methodology:** Fu-rong Fan, Shou-hong Ma, Hua Li, Jin-chun Li.

**Software:** Fu-rong Fan, Shou-hong Ma, Hua Li, Ming-wei Liu.

**Writing – review & editing:** Shou-hong Ma, Jin-chun Li, Ming-wei Liu.

**Visualization:** Hua Li, Yu Wen, Ming-wei Liu.

**Writing – original draft:** Jin-chun Li, Ming-wei Liu.

**Conceptualization:** Ming-wei Liu.

## References

[1] Shan F Long Y Qiu W. Autoimmune glial fibrillary acidic protein astrocytopathy: a review of the literature. Front Immunol. 2018;9:2802. 30568655 10.3389/fimmu.2018.02802 PMC6290896

[2] Long Y Liang J Xu H. Autoimmune glial fibrillary acidic protein astrocytopathy in Chinese patients: a retrospective study. Eur J Neurol. 2018;25:477–83. 29193473 10.1111/ene.13531

[3] Iorio R Damato V Evoli A. Clinical and immunological characteristics of the spectrum of GFAP autoimmunity: a case series of 22 patients. J Neurol Neurosurg Psychiatry. 2018;89:138–46. 28951498 10.1136/jnnp-2017-316583

[4] Ding J Ren K Wu J. Overlapping syndrome of MOG-IgG-associated disease and autoimmune GFAP astrocytopathy. J Neurol. 2020;267:2589–93. 32378036 10.1007/s00415-020-09869-2

[5] Yang X Xu H Ding M. Overlapping autoimmune syndromes in patients with glial fibrillary acidic protein antibodies. Front Neurol. 2018;9:251. 29755396 10.3389/fneur.2018.00251 PMC5932346

[6] Fang B McKeon A Hinson SR. Autoimmune glial fibrillary acidic protein astrocytopathy: a novel meningoencephalomyelitis. JAMA Neurol. 2016;73:1297–307. 27618707 10.1001/jamaneurol.2016.2549

[7] Greco G Masciocchi S Diamanti L. Visual system involvement in glial fibrillary acidic protein astrocytopathy: two case reports and a systematic literature review. Neurol Neuroimmunol Neuroinflamm. 2023;10:e200146. 37582612 10.1212/NXI.0000000000200146 PMC10427126

[8] Theuriet J Cluse F Gravier-Dumonceau A. Peripheral nervous system involvement accompanies central nervous system involvement in anti-glial fibrillary acidic protein (GFAP) antibody-related disease. J Neurol. 2023;270:5545–60. 37540278 10.1007/s00415-023-11908-7 PMC10576672

[9] Flanagan EP Hinson SR Lennon VA. Glial fibrillary acidic protein immunoglobulin G as biomarker of autoimmune astrocytopathy: analysis of 102 patients. Ann Neurol. 2017;81:298–309. 28120349 10.1002/ana.24881

[10] Zhu B Sun M Yang T. Clinical, imaging features and outcomes of patients with anti-GFAP antibodies: a retrospective study. Front Immunol. 2023;14:1106490. 37205100 10.3389/fimmu.2023.1106490 PMC10187143

[11] Zhang W Xie Y Wang Y. Clinical characteristics and prognostic factors for short-term outcomes of autoimmune glial fibrillary acidic protein astrocytopathy: a retrospective analysis of 33 patients. Front Immunol. 2023;14:1136955. 37350972 10.3389/fimmu.2023.1136955 PMC10282742

[12] Huang J Huang W Zhou R. Detection and significance of glial fibrillary acidic protein antibody in autoimmuneastocytopathy and related diseases. Ann Transl Med. 2023;11:288. 37090053 10.21037/atm-19-330 PMC10116425

[13] Carpo M Meucci N Allaria S. Anti-sulfatide IgM antibodies in peripheral neuropathy. J Neurol Sci. 2000;176:144–50. 10930598 10.1016/s0022-510x(00)00342-7

[14] Farah MH Dali CI Groeschel S. Effects of sulfatide on peripheral nerves in metachromatic leukodystrophy. Ann Clin Transl Neurol. 2024;11:328–41. 38146590 10.1002/acn3.51954 PMC10863914

[15] Lan W Li J Ai P. Autoimmune glial fibrillary acidic protein astrocytopathy: clinical analysis and review of 15 cases. Acta Neurol Belg. 2023;123:1465–79. 37079256 10.1007/s13760-023-02268-0 PMC10117260

[16] Dalmau J Graus F. Diagnostic criteria for autoimmune encephalitis: utility and pitfalls for antibody-negative disease. Lancet Neurol. 2023;22:529–40. 37210100 10.1016/S1474-4422(23)00083-2

[17] Cellucci T Van Mater H Graus F. Clinical approach to the diagnosis of autoimmune encephalitis in the pediatric patient. Neurol Neuroimmunol Neuroinflamm. 2020;7:e663. 31953309 10.1212/NXI.0000000000000663 PMC7051207

[18] Kimura A Takekoshi A Yoshikura N. Clinical characteristics of autoimmune GFAP astrocytopathy. J Neuroimmunol. 2019;332:91–8. 30991306 10.1016/j.jneuroim.2019.04.004

[19] Savaş M Tzartos J Küçükali CI. Glial fibrillary acidic protein (GFAP)-antibody in children with focal seizures of undetermined cause. Acta Neurol Belg. 2021;121:1275–80. 32333263 10.1007/s13760-020-01361-y

[20] Yang X Liang J Huang Q. Treatment of autoimmune glial fibrillary acidic protein astrocytopathy: follow-up in 7 Cases. Neuroimmunomodulation. 2017;24:113–9. 28922662 10.1159/000479948

[21] Wang WW Li M. Clinical features of autoimmune glial fibrillary acidic protein astrocytopathy in children: an analysis of 34 cases. Zhongguo Dang Dai Er Ke Za Zhi. 2023;25:67–72. 36655666 10.7499/j.issn.1008-8830.2208105 PMC9893831

[22] Fang H Hu W Jiang Z. Autoimmune glial fibrillary acidic protein astrocytopathy in children: a retrospective analysis of 35 Cases. Front Immunol. 2021;12:761354. 34880859 10.3389/fimmu.2021.761354 PMC8645641

